# The polypharmacology browser: a web-based multi-fingerprint target prediction tool using ChEMBL bioactivity data

**DOI:** 10.1186/s13321-017-0199-x

**Published:** 2017-02-21

**Authors:** Mahendra Awale, Jean-Louis Reymond

**Affiliations:** Department of Chemistry and Biochemistry, National Center of Competence in Research NCCR Chemical Biology and NCCR TransCure, University of Berne, Freiestrasse 3, 3012 Berne, Switzerland

**Keywords:** Target prediction, Polypharmacology, Drug–target interactions, Molecular fingerprints

## Abstract

**Background:**

Several web-based tools have been reported recently which predict the possible targets of a small molecule by similarity to compounds of known bioactivity using molecular fingerprints (fps), however predictions in each case rely on similarities computed from only one or two fps. Considering that structural similarity and therefore the predicted targets strongly depend on the method used for comparison, it would be highly desirable to predict targets using a broader set of fps simultaneously.

**Results:**

Herein, we present the polypharmacology browser (PPB), a web-based platform which predicts possible targets for small molecules by searching for nearest neighbors using ten different fps describing composition, substructures, molecular shape and pharmacophores. PPB searches through 4613 groups of at least 10 same target annotated bioactive molecules from ChEMBL and returns a list of predicted targets ranked by consensus voting scheme and *p* value. A validation study across 670 drugs with up to 20 targets showed that combining the predictions from all 10 fps gives the best results, with on average 50% of the known targets of a drug being correctly predicted with a hit rate of 25%. Furthermore, when profiling a new inhibitor of the calcium channel TRPV6 against 24 targets taken from a safety screen panel, we observed inhibition in 5 out of 5 targets predicted by PPB and in 7 out of 18 targets not predicted by PPB. The rate of correct (5/12) and incorrect (0/12) predictions for this compound by PPB was comparable to that of other web-based prediction tools.

**Conclusion:**

PPB offers a versatile platform for target prediction based on multi-fingerprint comparisons, and is freely accessible at www.gdb.unibe.ch as a valuable support for drug discovery.

**Graphical abstract:**
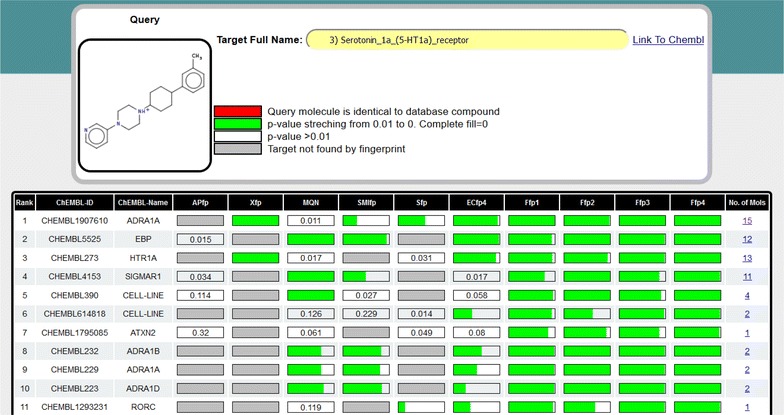
.

**Electronic supplementary material:**

The online version of this article (doi:10.1186/s13321-017-0199-x) contains supplementary material, which is available to authorized users.

## Background

The vast majority of small molecule drugs interact with multiple targets, a general phenomenon known as polypharmacology and a key parameter to be addressed in the course of drug development [1–3]. Many computational tools have been developed that exploit databases containing detailed structural information on the activity of small molecule drugs [4–8] and their protein targets [9] to predict the polypharmacology of any hit compound or drug candidate [10–37]. Several of these tools are accessible as target prediction websites (Table 1). Each of these websites returns a list of predicted targets based on similarity calculations using molecular fingerprints (fps) or on docking scores.

**Table 1:** Publicly accessible web-based target prediction tools

Website	Similarity method	Database	Ref.
www.gdb.unibe.ch	10 different fingerprints	ChEMBL 21	This work
http://www.pharmaexpert.ru/passonline/	Multilevel neighbourhoods of atoms (MNA) descriptors	WDI and ACD	[10]
http://www.dddc.ac.cn/tarfisdock/	Docking	PDTD	[12]
http://sea.bkslab.org	ECfp4	CHEMBL 16, WOMBAT, MDDR and StarLite	[14]
http://59.78.96.61/pharmmapper/	Receptor-based pharmacophore models	TargetBank, DrugBank, BindingDB, PDTD	[17]
https://cpi.bio-x.cn/drar/	Docking	PDB, DrugBank	[18]
www.cbligand.org/TargetHunter	ECfp6, ECfp4 and Openbabel FP2	ChEMBL 11 and PubChem bioassay	[22]
http://lilab.ecust.edu.cn/chemmapper/	Openbabel FP2, MACSS, SHAFT and USR	ChEMBL 14, BindingDB, DrugBank, KEGG and PDB	[23]
http://mips.helmholtz-muenchen.de/proj/hitpick	Circular fingerprint FCFP	STITCH	[24]
http://modlab-cadd.ethz.ch/software/spider/	CATS and MOE physiochemical descriptors	COBRA	[26]
www.swisstargetprediction.ch	Openbabel FP2 and Electroshape descriptors	ChEMBL 16	[28]
http://prediction.charite.de/index.php	ECfp4	ChEMBL, SuperTarget and BindingDB	[29]
www.dddc.ac.cn/tarpred	ECfp4	BindingDB	[33]
http://potentia.cbs.dtu.dk/ChemProt	Sfp	ChEMBL 14, BindingDB, DrugBank, PharmGKB, PubChem bioassay, WOMBAT, IUPHAR, CTD and STITCH	[37]

Ligand-based methods using fp comparisons are particularly versatile because they are applicable to any biological activity, i.e. the target may be a protein but also a cell line or a whole organism. Although some of these ligand based tools offer a selection of different fps, none of them permits the simultaneous use of more than one fp. Considering the fact that molecular similarity and therefore the predicted targets strongly depend on which fp is used for comparison, it would be highly desirable to predict targets using multiple fps simultaneously, provided that each fp would perform well individually in virtual screening and target prediction test cases. Herein we present the polypharmacology browser (PPB), a multi-fingerprint browser for target prediction which addresses this issue by performing target predictions searches using six different fps and four fused molecular fingerprints (Ffps) (Table 2). Similarities are measured using the city-block distance because this similarity measure is rapidly computed and therefore well-suited for web-based similarity searches in large databases [38–45]. PPB searches through 2.7 M ligand-target interactions extracted from ChEMBL 21 and generates a list of predicted targets, each linked to the lists of known actives used for the prediction. PPB validation is presented for 670 drugs of known polypharmacology as well as in a predictive application of off-targets for a recently reported inhibitor of transient receptor potential vanilloid 6 (TRPV6) [46]. PPB is freely accessible at www.gdb.unibe.ch and works on computers, tablets and phones.

**Table 2:** Molecular fingerprints used for target prediction with PPB

Name	Description	Ref.
APfp	21-D atom-pair fingerprint, perceives molecular shape	[43]
Xfp	55-D atom category extended atom-pair fingerprint, perceives pharmacophores	[43]
MQN	42-D Molecular Quantum Numbers, scalar fingerprint counting atoms, bonds, polarity and ring features, perceives constitution, topology and molecular shape	[38]
SMIfp	34-D scalar fingerprint counting occurrence of characters in SMILES, perceives rings, aromaticity, and polarity	[42]
Sfp	1024-D binary daylight type substructure fingerprint, perceives detailed substructures	[49]
ECfp4	1024-D binary circular extended connectivity fingerprint, perceives detailed substructures and pharmacophores	[50]
Ffp1	Fusion fingerprint, Xfp + SMIfp + Sfp	This work
Ffp2	Fusion fingerprint, Xfp + MQN + SMIfp	This work
Ffp3	Fusion fingerprint, Xfp + SMIfp + Sfp + ECfp4	This work
Ffp4	Fusion fingerprint, Xfp + MQN + SMIfp + Sfp + ECfp4	This work

## Methods

### Dataset

**Fig. 1:**
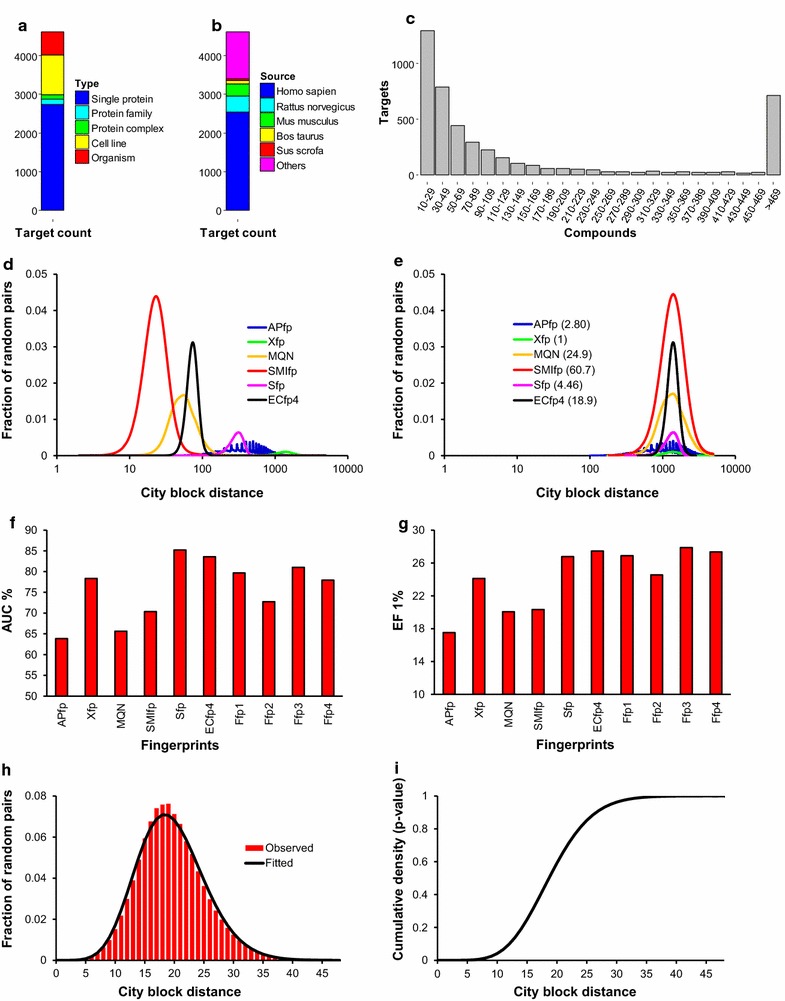
Overview of the data used for constructing PPB. Distribution of **a** target type as defined in ChEMBL and **b** source of targets. **c** Distribution of targets as per number of associated bioactive compounds. **d** Histogram of city block distances (log scale) calculated for 50 million random pairs of compounds from ChEMBL 21 using six molecular fingerprints. **e** APfp, MQN, SMIfp, Sfp and ECfp4 fingerprints were scaled with respect to Xfp to adjust to the value of the most frequent distance. Scaling factors are shown in parentheses. **f**, **g** Enrichment of 40 set of DUD actives from corresponding decoys set by six different fingerprints (APfp, Xfp, MQN, SMIfp, Sfp and ECfp4) and four similarity fusion methods (Ffp1–4). City block distance was used as sorting function. Data is represented as average of **f** Area under ROC curve and **g** Enrichment factor at 1% of screen database for 40 targets from DUD. **h**, **i** Example of *p* value calculation. **h** Observed (*red*) and fitted (*black*) random distance distributions for the muscarinic acetylcholine receptor M1 (CHRM1, CHEMBL216) in MQN fingerprint space. City block distances were calculated for 1788 ligands of CHRM1 with respect to random compounds from ZINC database. Negative binomial distribution was used for curve fitting. **i** Cumulative density plot indicating area under fitted curve in **h**

We analyzed ChEMBL 21 and constructed the target database containing 4613 groups of at least 10 bioactive molecules with documented activity against the same biological target. Briefly, target database was constructed as follows: initially all targets along with their ligands were retrieved from the ChEMBL version 21. For each target we retained the compounds having IC_50_, EC_50_, GI_50_, *K*_i_, *K*_D_, or potency value of ≤10 µM or percent inhibition of >50%. All molecules were processed as non-stereo SMILES and ionized at pH 7.4 using an in-house developed Java program utilizing the JChem chemistry library from ChemAxon Pvt. Ltd. Afterwards, duplicate molecules were removed in the context of each target. Finally, targets with at least 10 bioactive compounds were retained in database. In total, these 4613 targets represent 871,673 unique bioactive compounds and 2.7 M ligand-target interactions. Of these targets, 60% are single protein type, 55% are human targets, and 45% have less than 50 bioactive compounds (Fig. 1a–c).

### Fingerprints

For each of the 871,673 selected ChEMBL compounds we computed each of the six fingerprints described in Table 2. In short, APfp, Xfp, MQN and SMIfp were calculated by using an in house developed Java program utilizing various calculator plugins from JChem chemistry library, in particular TopologyAnalyserPlugin to determine shortest topological path for atom-pair, HBDAplugins to determine hydrogen bond donor and acceptor atoms, and MajorMicrospeciesPlugin to adjust the ionization state of molecules. The detailed procedure for the generation of these fingerprints can be found in the respective publication [38, 42, 43]. For Sfp, a daylight type 1024-bit hash fingerprint was computed using the ChemicalFingerprint class of the JChem library. The 1024-bit extended connectivity fingerprint (ECfp4) was calculated with bond diameter of 4 using ECFP class of the JChem library. The source codes for computation of fingerprints are freely available for download at www.gdb.unibe.ch.

### Fused fingerprints (Data fusion)

To generate additional molecular fingerprint descriptions we further investigated data fusion between different combinations of these fingerprints [47]. Since we aimed at using the city-block distance (Eq. 1) as similarity measure, we scaled each fingerprint by analyzing the distance distribution of 50 M random pairs of compounds in each fingerprint space, and scaled values to adjust the most frequently occurring distance in each fp to the value for Xfp (Fig. 1d/e). We then performed enrichment studies of ligands against decoys in the directory of useful decoys (DUD) [48] and evaluated the average performance of 57 different combinations of the scaled fingerprints in terms of area under the curve (AUC) and enrichment factor at 1% screening in the receiver operator characteristic (ROC) curves (data not shown). We selected the four fusion fingerprints Ffp1–Ffp4 (Table 2) due to their good performance in this enrichment study (Fig. 1f/g), and computed the corresponding Ffp1–4 values for the 871,673 ChEMBL compounds.

1 $${CBD_{A,B} = \sum\limits_{j = 1}^{K} {\left| {A_{j} - B_{j} } \right|} }$$

### *p* value calculation

Each target prediction for a given query molecule is based on the city-block distance between the query molecule and the closest member of a group of compounds associated with this target. A *p* value can be computed for each prediction as the degree of randomness of the observed city block distance [51] and therefore the probability that the corresponding query–target association occurs at random. To compute the *p* value, we generated a random distance distribution for each of the 4613 targets in each of the ten fingerprint spaces by computing distances between the ChEMBL compounds associated with the target and randomly selected molecules from the ZINC database [52], taken as representative molecules of screening compounds for which target predictions might be carried out (Fig. 1h). For each target up to 1 M random pairs were considered. We then fitted each of the 46,130 distance distributions using a negative binomial distribution function, and generated the corresponding cumulative density functions giving the *p* value as a function of the city block distance (Fig. 1i). The choice of a negative binomial distribution function was based on the discrete nature of the city-block distance. As the *p* value calculation is specific for each target protein and fingerprint space it can only be used in this context, and should not be used to compare molecules from different targets or fingerprint spaces. The curve fitting was carried out using the R statistical package version 3.2.5 using the “fitdistrplus” library with default maximum likelihood method for parameters estimation.

### Validation set

The validation set containing 670 drugs and their targets annotation was created as follows: (a) compounds labelled as approved drug or drug in clinical trial were extracted from ChEMBL database, (b) for each drug, target list was constructed by comparing SMILES string of drug to SMILES of bioactive compounds of targets used in the PPB. When the SMILES string was matched, corrosponding PPB target was added to known target list for the drug and (c) retained the drugs with ≤20 targets in the list.

### The PPB web-interface

Given a query molecule, PPB computes each of the 10 fps for this compound, sorts the 871,673 ChEMBL compounds by city-block distance to the query using each fp independently, and collects a predefined number of compound associated targets in each case. The *p* value for each target is calculated from the nearest neighbor found for that target. Finally, PPB merges the different target lists.

The graphical user interface of PPB starts with an initial page wherein the user can input the structure of a query molecule using the JavaScript based JSME molecular editor (http://peter-ertl.com/jsme/) [53]. The structure can be drawn or copy-pasted in SMILES or sdf file format. An option is available to extract the query molecule from the Protein Data Bank (PDB) using the PDB id of the protein–ligand complex of interest (the PDB ligand data was downloaded from http://ligand-expo.rcsb.org/ website in March 2016 and stored on our web server, and will be updated once a year). The option “No. of targets” allows the user to input the number of targets to be returned by each fp. By default this parameter is set to 20. Following the entry of a query molecule, the target search can be initiated by clicking on the “Submit” button. Typically, execution of a search takes less than 1 min.

**Fig. 2:**
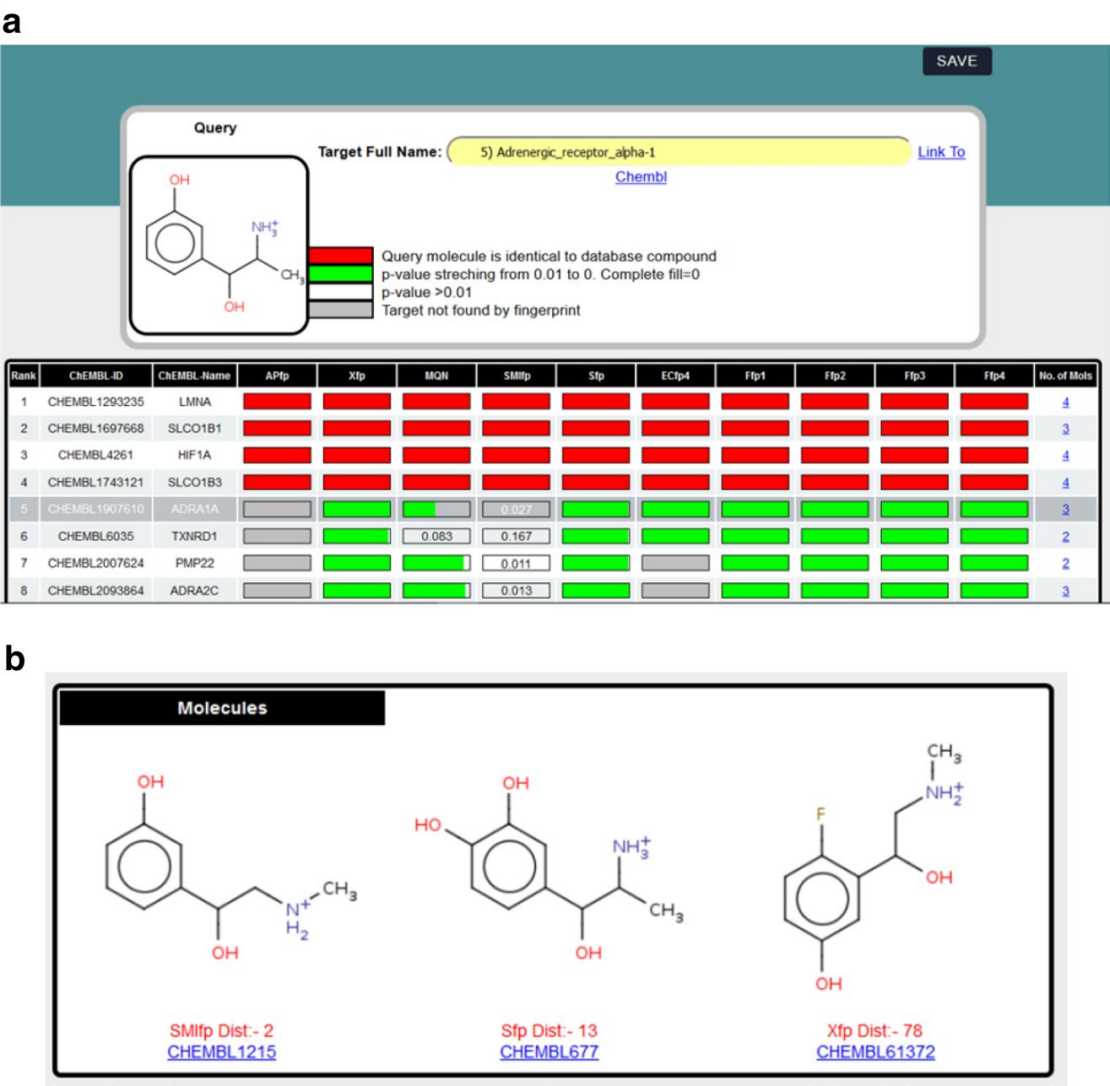
PPB web-browser. **a** Result panel displaying the PPB predicted targets for the drug metaraminol. **b** List of molecules for the target selected in the result panel (*row 5* ADRA1A)

The target prediction results are presented as list of targets annotated with a probability bar for each fp (Fig. 2a). The *p* value is shown by a green bar of decreasing length up to *p* = 0.01, with values above 0.01 written as number in the white probability bar. A grey bar indicates that the target was not found, and a red bar indicates that a molecule with distance = 0 (usually the identical molecule) is present in the ChEMBL reference list. Each target is labelled with its short name and ChEMBL target id, and the number of compounds retrieved by the browser is indicated in the last column of the table. In the initial display the list of targets is sorted by frequency of occurrence and sum of *p* values across the 10 fps, which defines the target rank (first column). The list can also be sorted by ChEMBL target id, target name, and by the number of selected compounds by clicking on the corresponding column heading.

Clicking on the number of selected compounds per target (furthest right column) opens a new tab displaying the structures of these compounds labelled with fingerprints, city-block distance and ChEMBL compound id (Fig. 2b). The selection of a row in the table displays the full name of the target in the “Target name” field, which is an active link to the parent ChEMBL database to obtain further information on this target. The result (targets and molecules) can be stored as text file using the “save” button provided in each window.

## Results and discussion

**Fig. 3:**
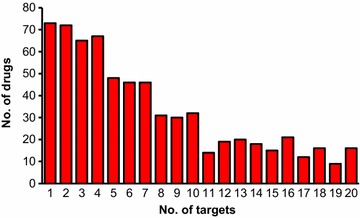
Distribution of 670 ChEMBL compounds labelled as drug in clinical trial or as approved drug used in validation study according to number of associated targets

The performance of PPB was evaluated by challenging its ability to recall the known targets of 670 compounds labelled as approved drug or drug in clinical trial and annotated with up to 20 targets in ChEMBL (4794 drug–target interactions). Prior to evaluation these 670 drugs were removed from our target compound database. Among these drugs 71% had less than 10 associated targets and the remaining 29% had 10–20 associated targets (Fig. 3).

**Fig. 4:**
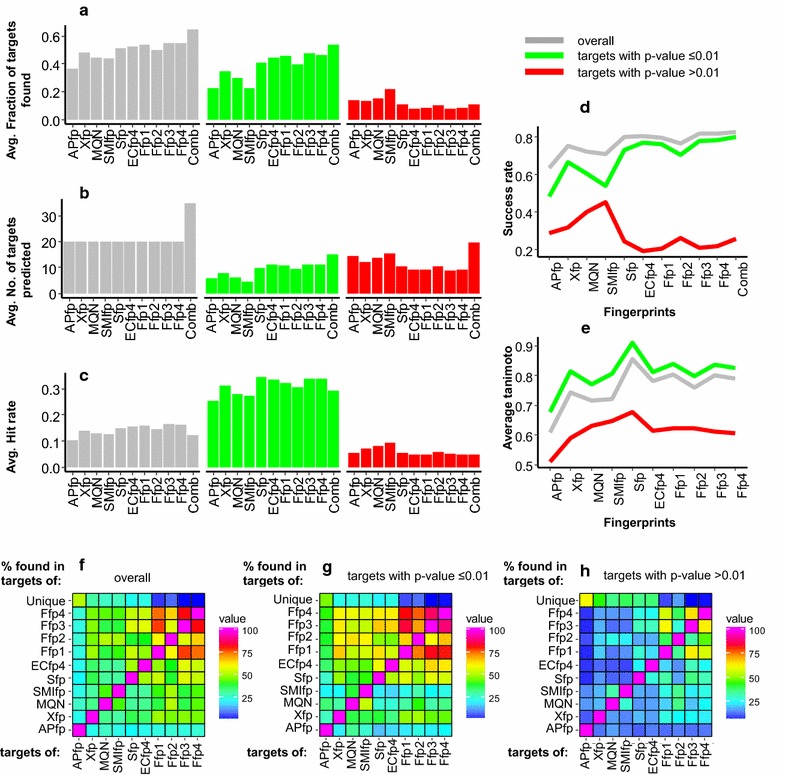
Recovery statistics of targets of 670 drugs by various fingerprints and combined method used in PPB. The *bar* plots shows an average, **a** fraction of known targets found, **b** number of targets predicted and **c** hit rate calculated for 670 drugs (see Additional file 1: Fig. S1). For each drug analysis was performed at three different levels considering all targets (*grey*), targets with *p* value of ≤0.01 (*green*) and targets with *p* value of >0.01 (*red*). **d** Success rate for finding at least 1 known target of drugs among top 5 predicted targets by each method. **e** Average Tanimoto coefficient of binary substructure fingerprint between the query and the most similar bioactive ligand associated with correctly predicted known targets in the results list. **f–h** Percentages of targets of one fingerprint found by another fingerprint and percentages of targets unique to this fingerprint, considering three different targets lists as mentioned before

The 670 different searches were performed using PPB with the default search settings. The predicted targets were analysed for each fp considering (a) all targets, (b) targets with *p* value ≤0.01 and (c) targets with *p* value >0.01 (Fig. 4; Additional file 1: Fig. S1). When combining the results of the different fps (last column, “comb” in Fig. 4a–d) we only considered the targets voted by at least 2 different fps. For each drug, we calculated the fraction of known targets in the results list, the total number of predicted targets and the hit rate (ratio of known targets found to predicted targets).

In terms of the fraction of correctly predicted known targets (Fig. 4a) the fusion fingerprints Ffp1, Ffp3 and Ffp4 showed the highest average value, followed by the binary fingerprints Sfp and ECfp4, the pharmacophore fingerprint Xfp, and finally MQN, SMIfp and APfp. To evaluate if the performance of these fingerprints significantly differ from one another or not, the student *t* tests (at confidence interval of 0.95) were performed for all the possible pairs of fingerprints (Additional file 1: Tables S1 and S2). Although most of the individual fingerprint pairs show significant differences in performance, no significant differences were found between performance of Sfp, ECfp4, Ffp1 and Ffp2. Overall the performance trend followed the complexity of each fingerprint and highlights that detailed structural encoding tends to increase prediction performance.

These single fp approaches were outperformed by using the combination of all 10 results lists (combined method), however at the expense of a relatively low hit rate resulting from checking a larger number of targets for each drug as compared to individual fps (Fig. 4b/c). The combined method also showed the highest success rate for finding at least one known target among the 5 top predicted targets for each fp (Fig. 4d). For all methods except SMIfp the predicted target list with *p* value ≤0.01 showed a significantly greater chance of success compared to the target lists with *p* value >0.01. The importance of a low *p* value for target prediction was particularly striking with the fused fingerprints Ffp1–4 and the combined method, for which more than 75% of all correct predictions originated from predicted targets with *p* value ≤0.01.

Although several of the fps were not statistically different in terms of performance, the pairwise overlap between predicted targets by each of the 10 different fps showed that on average less than 45% of targets were common between any two fps (Fig. 4f). Furthermore each fp retrieved a significant number of unique targets which are not found by any other fp, further highlighting the utility of each fp. Interestingly, APfp and Xfp, which perceive the shape and pharmacophore patterns in molecules, returned the highest percentages of unique targets (52 and 31% respectively). For fused fingerprints Ffp1–4 the percentages of unique targets were relatively low (3–10%) due to the considerable overlap among themselves.

A similar analysis performed by categorizing targets according to their *p* values (Fig. 4g/h) showed that the pairwise overlap between high confidence (low *p* value) targets of different fingerprints were significantly higher (on average 47% overlap) as compared to low confidence targets (high *p* value, on average 24% overlap). This can be explained by the fact that at high *p* values the structural similarity between a query and its nearest neighbour compound associated with the target becomes less obvious and difficult to capture (Fig. 4e).

### Prediction of off-targets of a new TRPV6 inhibitor

**Fig. 5:**
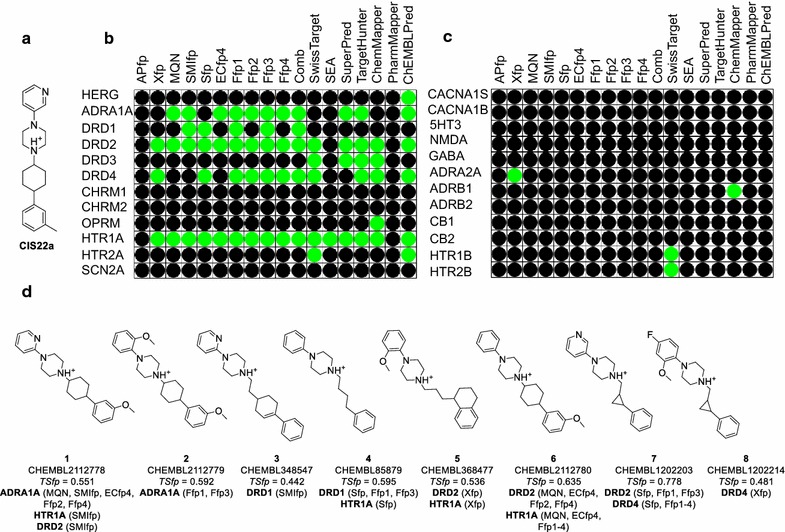
Prediction of targets of CIS22a using PPB and comparison with other web-based tools. In case of ChEMBLPred target prediction models (10 μM) were downloaded from ChEMBL website and implemented locally using RDkit and python. **a** Structure of CIS22a. **b** Confirmed side targets of CIS22a. **c** Targets detected with no significant binding affinity for CIS22a. Targets which were found and not found by the fingerprints used in PPB and external web-based tools are indicated with *green* and *black dots*, respectively. For external web-based tools, at the maximum top 30 predicted targets were considered. The prediction performance of ChemProt, HitPick, TarPred, SPiDER, PASS, TarFishDock and Drar web based tools listed in Table 1 are not shown due to technical failures or no applicability in the context. **d** Structure, ChEMBL id and tanimoto coefficient for bioactive compounds which linked the targets to CIS22a, indicated with the name of fingerprints in *parentheses*. Target full names: Adrenergic α1A (ADRA1A) and α2A (ADRA2A) receptor, Adrenergic β1 (ADRB1) and β2 (ADRB2) receptor, Cannabinoid 1 (CB1) and 2 (CB2) receptor, Voltage dependent L- (CACNA1S) and N-type (CACNA1B) Ca^2+^ channel, Cholinergic muscarinic receptor 1 (CHRM1) and 2 (CHRM2), Dopamine receptor subtypes D1-4 (DRD1-4), Gamma aminobutyric acid receptor (GABA), 5-hydroytryptamine receptor 3 (5-HT3), 5-hydroytryptamine receptor 1A (HTR1A), 1B (HTR1B), 2A (HTR2A) and 2B (HTR2B), Voltage gated potassium channel subfamily H member 2 (HERG), *N*-methyl-d-aspartate receptor (NMDA), µ opioid receptor (OPRM), voltage gated Na^+^ channel (SCN2A)

We recently reported the identification of CIS22a (Fig. 5a) as the first potent and selective inhibitor of TRPV6, a transmembrane calcium channel overexpressed in breast and prostate cancer [46]. We tested the polypharmacology of this inhibitor for 24 out of 44 targets present in the “safety screen” panel of Cerep Pvt. Ltd (Fig. 5b/c). To assemble this target list we first inspected the PPB results list and chose five targets selected by multiple fps considering in each case the target from human and rat origin as the same target. These were the adrenergic α1A receptor (ADRA1A), the dopamine receptor subtypes D1 (DRD1), D2 (DRD2), and D4 (DRD4), the 5-hydroxytryptamine receptor 1A (HTR1A). We then added further subtypes of these five targets as well as all ion channels present in the safety screen panel, resulting in a list of 17 GPCRs and seven ions channels.

In-vitro profiling showed that CIS22a bound significantly (>50% inhibition at 10 µM) to 12 targets of the 24 selected targets (Fig. 5b). Five of these targets (ADR1A, DRD1, DRD2, DRD4 and 5HTR1A) were proposed by multiple fps in the PPB. Only two of the Ffps (Ffp1 and Ffp3) and the combined method were able to predict all of the five targets, illustrating the usefulness of similarity fusion methods and combined data analysis. The analysis of bioactive compounds which linked these five targets to CIS22a showed that the linking compounds were shared by different fps and closely related targets (**1–8** in Fig. 5d). Interestingly, the linking compounds for three targets (DRD2, DRD4 and HTR1A) suggested by Xfp were not shared by any other fps, probably because of their relatively low substructure similarity to the query compound. On the other hand Xfp predicted an activity on ADRA2A, which was not confirmed experimentally (only 31% inhibition at 10 µM).

For comparison we successfully ran target predictions for CIS22a using six of the fourteen target prediction web-based tools listed in Table 1. Results comparable to PPB were obtained with SwissTarget, SuperPred, TargetHunter, ChemMapper and ChEMBLPred. On the other hand, SEA only returned a single, correct target, and PharmMapper did not predict any of the tested targets.

## Conclusion

The PPB web tool features a unique, intuitive and exhaustive search platform for target prediction. The comparative view of target list from various fingerprint spaces provides a simple yet efficient way for selection of targets by consensus voting. PPB provides a valuable support to drug discovery projects.

## Additional file

**Additional file 1.** Figure S1: Recovery statistics of targets of 670 drugs by various fingerprints and combined method used in PPB. Tables S1 and S2: Student t-test significance values for pair wise comparison of target prediction performance of all the fingerprints used in PPB.
