# Supplementary material for: The polypharmacology browser: a web-based multi-fingerprint target prediction tool using ChEMBL bioactivity data
Source: J Cheminform. 2017 Feb 21;9:11. doi: 10.1186/s13321-017-0199-x (PMC5319934; doi:10.1186/s13321-017-0199-x)
Supplement: Supplementary file 1 — Additional file 1. Figure S1: Recovery statistics of targets of 670 drugs by various fingerprints and combined method used in PPB. Tables S1 and S2: Student t-test significance values for pair wise comparison of target prediction performance of all the fingerprints used in PPB. [file 13321_2017_199_MOESM1_ESM.pdf]

Supporting Information for:

The polypharmacology browser: a web-based multi-fingerprint target prediction tool using ChEMBL bioactivity data

Mahendra Awale and Jean-Louis Reymond*

Department of Chemistry and Biochemistry, University of Berne, Freiestrasse 3, 3012 Berne Switzerland; e-mail: jean-louis.reymond@ioc.unibe.ch; FAX: +41 31 631 80 57

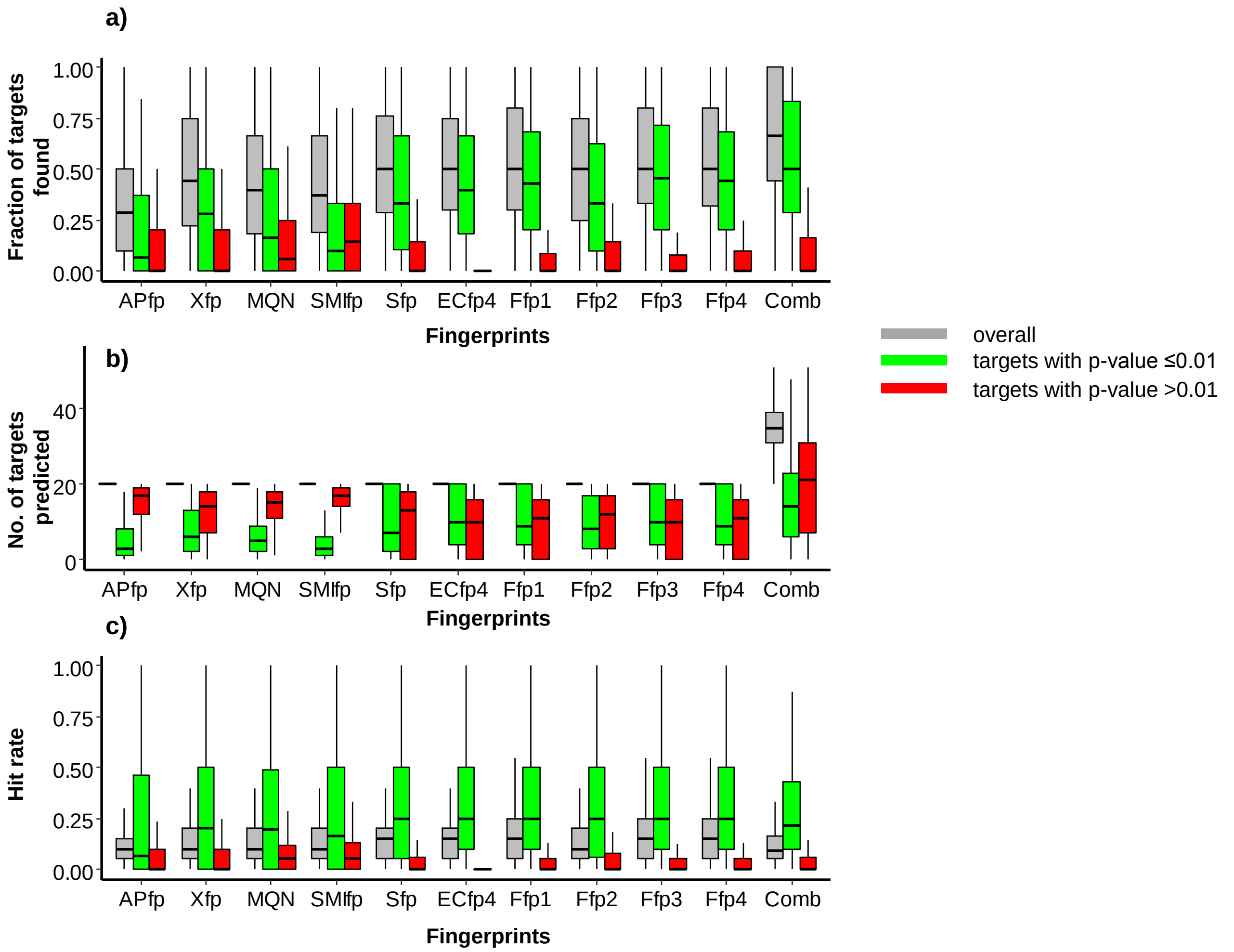

Figure S1. Recovery statistics of targets of 670 drugs by various fingerprints and combined method used in PPB. The box plots shows distribution of **(a)** fraction of known targets found, **(b)** number of targets predicted and **(c)** hit rate calculated for each of the 670 drugs (outliers are not shown). For each drug analysis was performed at three different levels considering all targets (grey), targets with p-value of ≤0.01 (green) and targets with p-value of >0.01 (red).

Table S1. Student t-test significance values for pair wise comparison of target prediction performance of all the fingerprints used in PPB.

Fp/Fp[a)]	APfp	Xfp	MQN	SMIfp	Sfp	ECfp	Ffp1	Ffp2	Ffp3	Ffp4	Comb
**APfp**	1	0	0	2.00E-05	0	0	0	0	0	0	0
**Xfp**	0	1.00	0.05	0.02	0.08	0.02	0.00	0.25	7.00E-05	0.00	0.00
**MQN**	0	0.05	1.00	0.72	2.00E-04	2.00E-05	0.00	0.00	0.00	0.00	0.00
**SMIfp**	2.00E-05	0.02	0.72	1.00	4.00E-05	0.00	0.00	0.00	0.00	0.00	0.00
**Sfp**	0	0.08	2.00E-04	4.00E-05	1.00	0.61	0.13	0.54	0.02648	0.04	0.00
**ECfp**	0	0.02	2.00E-05	0.00	0.61	1.00	0.31	0.26	0.08496	0.13	0.00
**Ffp1**	0	0.00	0.00	0.00	0.13	0.31	1.00	0.03	0.49	0.63	0.00
**Ffp2**	0	0.25	0.00	0.00	0.54	0.26	0.03	1.00	0.00	0.01	0.00
**Ffp3**	0	7.00E-05	0.00	0.00	0.03	0.08	0.49	0.00466	1.00	0.83	0.00
**Ffp4**	0	0.00	0.00	0.00	0.04	0.13	0.63	0.01	0.83	1.00	0.00
**Comb**	0	0	0	0	0	0	0	0	0	0	1.00

[a)] t-test was performed using distribution of "Fraction of targets found" values considering all targets (Figure S1, grey). Significance values >0.05 are highlighted in red.

Table S2. Student t-test significance values for pair wise comparison of target prediction performance of all the fingerprints used in PPB.

Fp/Fp[a)]	APfp	Xfp	MQN	SMIfp	Sfp	ECfp	Ffp1	Ffp2	Ffp3	Ffp4	Comb
**APfp**	1	0	4.00E-05	0.94436	0	0	0	0	0	0	0
**Xfp**	0	1.00	0.00	0.00	0.00	0.00	0.00	0.01	0	0.00	0.00
**MQN**	4.00E-05	0.00	1.00	5.00E-05	0	0	0.00	0.00	0.00	0.00	0.00
**SMIfp**	0.94436	0.00	5.00E-05	1.00	0	0.00	0.00	0.00	0.00	0.00	0.00
**Sfp**	0	0.00	0	0	1.00	0.03	0.01	0.63	0.00034	0.00	0.00
**ECfp**	0	0.00	0	0.00	0.03	1.00	0.63	0.01	0.14773	0.35	0.00
**Ffp1**	0	0.00	0.00	0.00	0.01	0.63	1.00	0.00	0.34	0.66	1.00E-05
**Ffp2**	0	0.01	0.00	0.00	0.63	0.01	0.00	1.00	4.00E-05	0.00	0.00
**Ffp3**	0	0	0.00	0.00	0.00	0.15	0.34	4.00E-05	1.00	0.60	0.00
**Ffp4**	0	0.00	0.00	0.00	0.00	0.35	0.66	0.00	0.60	1.00	5.00E-05
**Comb**	0	0	0	0	0	0	1.00E-05	0	0.00042	5.00E-05	1.00

[a)] t-test was performed using distribution of "Fraction of targets found" values considering targets with p-value of ≤0.01 (Figure S1, green). Significance values >0.05 are highlighted in red.

Student t-test calculation

Student t-test was performed using R statistical software package version 3.2.5 with the default parameter setting (0.95 Confidence interval). The input to the student t-test was two different lists (corrosponding to two different fingerprints) of 670 fraction of target found values. The t-test was repeated for all the possible pairs of fingerprints used in PPB considering all correctly predicted targets (Table S1) and targets with p-value of ≤0.01 (Table S2).
